# Supplementary material for: Patterns of Intron Gain and Loss in Fungi
Source: PLoS Biol. 2004 Nov 30;2(12):e422. doi: 10.1371/journal.pbio.0020422 (PMC532390; doi:10.1371/journal.pbio.0020422)
Supplement: Table S1 — Also available at http://genes.mit.edu/NielsenEtAl/. (4.3 MB ZIP). [file pbio.0020422.st001.zip › NielsenEtAl/html/614.html]

AN3157.1.NCU09642.1.MG09838.1.FG04242.1


```
 CLUSTAL W (1.82) Multiple Sequence Alignments - Introns Inserted


Sequence 1: NCU09642.1	799 aa
Sequence 2: MG09838.1	800 aa
Sequence 3: FG04242.1	789 aa
Sequence 4: AN3157.1	755 aa
Alignment Length: 819 aa
Number Identitical Residues: 306 aa
Alignment Score (without introns) 15655


MG09838.1 	MSSGDNNSPGGSSRPQAVSRNSTNNASGRNNHAPANPSGLRNVVDPSEDASATN---NNG
NCU09642.1	MPSRPSSLRSIPGPLNSNPPNAQAPTGPTHDHAPAHPSGLRQSYTVSSSYSSTSDFERDG
FG04242.1 	MANRPSHSRTNSSTDHRPAQPSTLRQTVNRNQNDASATGNPAPPESPGHPQGAT-----I
AN3157.1  	-MTESDHTRSLRDRIIELFRPPASVTFLPRMTVHNNTMGDFDESAGSNSSQVDS------
          	  .  .      .        .       .      . *       .   .  .      

MG09838.1 	DAGPSEPRSPTRNAASETTSLLRGVHDDHHPHEGQCNHGTFSPRPSSPTSILSSEPSFAT
NCU09642.1	DEDSGAGPSNDNPVRSVTPNERTGLLSQERGHGTFTDARTVSPSNASHADSLPGFPFDAP
FG04242.1 	DEIFANESTPLIDGNNQRPSQRRG--SVHPAHPGVCSHGTFSPRPMSPTLTMRSTDEDGS
AN3157.1  	-------RTPLLGSYDRR--------EPPCGMGNSCNHGTFTPRVESDDWRTNAMPEYGG
          	        :      .          .         .  *.:*   *      .    . 

MG09838.1 	SE-----SSTSRLFENFLGTITGNSND--PDWRRKLARRIRSKKMHTSQELAREHGIRDS
NCU09642.1	TDTDLDFSPYNSESPNGEGPASGKPKHKRKSWKRQISSHITTQKMHTSRDLARRHGVKDT
FG04242.1 	ET-----AGSGTHIPVLDNAITTIVGH--EDWKKWLKKRMRTKTMGHSSVLAEQAGIEDT
AN3157.1  	ER-----YPIGSTSRRSTSAAPS-------------AQGLENFPAGASTPLT----IKYS
          	          .       .. .                 : .     *  *:    :. :

MG09838.1 	PLM~YLSYYIPCLNWMSQYKRSYLKGDLIAAVTIAGMYLPMALSLADNLAHVPPINGLYG
NCU09642.1	PGM~YLSYYLPVLVWAREYSWSYFKGDFIAALTVAGMYVPMALSLADNLAHVPAINGLYS
FG04242.1 	AFM2YMAYYIPCLNWMRQYKVSYLRGDLIAAVTMASFYLPMALSLAANLAHVPPIHGLYA
AN3157.1  	KSQ2YISYYIPFFTWISQYRLSYLRGDICAALTVASIYIPMGLSLSN-IAHAPPINGLYS
          	    *::**:* : *  :*  **::**: **:*:*.:*:**.***:  :**.*.*:***.

MG09838.1 	FVFQPFVYALLGSSPQMMVGPEAAGSLLVGSVVKSTLDRTSGTPSSMGGGAENDAELQAK
NCU09642.1	FVWNPLIYALLGSCPALIVGPEAAGSLLVGAAVKSSVD--------LGRGGDEDRVLHAK
FG04242.1 	FVFNPFIYALLGSCPQMVVGPEAAGSLLVGTVVKQNVG---------SGDDEDNDMLHAQ
AN3157.1  	FVLQPFIYAILGSSPLLVVGPEAAGSLLTGAIVKESVN--------QGSSGEDDEAANAI
          	** :*::**:***.* ::**********.*: **..:.         . . :::   :* 

MG09838.1 	VCGIVGGIAAATVFIAGLTRLGFLDSVLSRPFLRGFISAIGVVIGVDQLIPELGLNKLAA
NCU09642.1	ICGIVAGMAGAMVFAAGVARLGFLDSVLSRPFLRGFISAIGVVIAVDQLIPELGLNHLVA
FG04242.1 	ICGIVAGMAGAMVVIAGLARLGFMDSVLSKPFLRGFISAIGFVIAVDQLIPELGLAELAD
AN3157.1  	VVGVATALAGLMILVAGIFRLGFLDNVLSRPFLRGFITAIGFVIFVDQFIPELGLAAEAK
          	: *:. .:*.  :. **: ****:*.***:*******:***.** ***:******   . 

MG09838.1 	ATPAVGHGSSMDKLRFMFDNLDQVHKMTALVAGVSFTVIMVAR2ETKRRLQPRYPGVAYV
NCU09642.1	KHPGIGHGSSVEKLRFILGNLDKVHKLTAVVAGTSFVVIMVCR~EIKRRMQPRYPGVAYV
FG04242.1 	K-AGVSHGSPVEKIRFIIGNVDKAHGLTFAIAGISFLVIMICR2ELKNRLQPRYPGVAYI
AN3157.1  	A-AGVTHGSTVDKLLFIIRNITHSHSLTAAVAFTSFAIIMVFR2TLKKLLQPRYPQVIYF
          	  ..: ***.::*: *:: *: : * :*  :*  ** :**: *   *. :***** * *.

MG09838.1 	PDRFIVVVLSAWLAYYFEWEKHGVQILGTVKAASGRPFTFRWPFQPSHVVQIRDAMGTSF
NCU09642.1	PDRFLVVVLTSFLAYWFQWDKKGVAVLGTVEAASHQTFKFRWPFQPSNMQLIRDTMGTSF
FG04242.1 	PDRFIVVVISAILCWQLDWENQGVEILGIVKAANGQLLAFQWPFKLQHMPHIRSAMSTSF
AN3157.1  	PDRILVVVISAVLTQQLRWHEQGLEVLGPTKNSGNGVFNFEWPFQPKYAEYLRTALSTSF
          	***::***::: *   : *.::*: :** .: :.   : *.***: .    :* ::.***

MG09838.1 	LLAMLGFFESSVAAKSLG--DQTIFPDIQLSPNRELIALGAANLLGSCFMSLPAFGGYGR
NCU09642.1	LIALLGFFESSVAAKSLS--SSEPFAGIQLSANRELVALGVANFVGGCFMSLPAFGGYGR
FG04242.1 	LIALLGFFESSVAAKSLG--SSETIQGIQLSANREMIALGIANMVGGCFMSLPAFGGYGR
AN3157.1  	IIAILGFFESSVAAKGLTGVASVGVQGMQVSPNREMVALGFANVVGGCFMSLPAFGGYAR
          	::*:***********.* .  .  . .:*:*.***::*** **.:*.***********.*

MG09838.1 	SKVNRQTGGKTPMSSLFVSFIALMVILFGLRLFYYLP0KPVLSSLISVVAWSLIEECPHD
NCU09642.1	SKVNKATGGKTPMSSILLSLLTLFTILFLLPYLYYLP0KPVLSALISVVAWSLIEECPHD
FG04242.1 	SKVNKSTGGKSPASSMFLSIISLLSIFFLLPYFYYLP0KPVLSSMISVVAYSLIEEAPHD
AN3157.1  	SKVNASTGARSPMSNICLSVVAIICIMVLLPYLYYLP0KAVLSSMISVVAYSLIEECPHD
          	****  **.::* *.: :*.:::: *:. *  :**** *.***::*****:*****.***

MG09838.1 	IYFFLRIRAWQELSLMFLIVLTTIFFSLNMGIAIGIGISLLLVIRNSTRPHIQILGRIPG
NCU09642.1	ITFFLRLRAWPELALMAIILLSTIFFSLTLGMAIGVGLSLLQVIRNATRPRIQILGRIPG
FG04242.1 	ISFFLKIRGWTELGLMAVIFLATMFYSLTLGMAFGVGISMLMVIKHSTRPRIQILGRIPG
AN3157.1  	LAFFIRLRGWSELALMLLIFLSTIFYSLELGIALGMGLSVLILIRHSTKPRIQILGKVAG
          	: **:::*.* **.** :*.*:*:*:** :*:*:*:*:*:* :*:::*:*:*****::.*

MG09838.1 	TNRFENAEAH---PERLEFVEGCLIVKIPEPLTFANTGQLKNRLRRLELYGTNKAHPALP
NCU09642.1	TNRFENAEFFTDSPDRLEFIEGCLIVKIPEPLTFANTGELKSRLRRLERYGTNEAHPALP
FG04242.1 	TNRFENAEGD---KASLEFVEGCLIVKIPEPLTFANTGELKSRLRRLELYGTSKAHPALP
AN3157.1  	SDRFDNAELH---PENVESVEGALVVKIPEPLTFANTGDLKNRLRRLEMYGTNRAHPSMP
          	::**:***        :* :**.*:*************:**.****** ***..***::*

MG09838.1 	RLRAQVQNKNVVFDVHGVTSMDGSGTQVLEEIVRGYRDRGVRVFFSRLPSRDSSPVWKLM
NCU09642.1	RLRQEDSNKNIIFDVHGVTSMDGSGTQVLEEIVRGYRERGVRVFFSRVPGGKDSEVWKMM
FG04242.1 	RLRHADMNRNVIFDIHGVTSMDGSGTQVLAEIVRNYTERGVRVYFSRCPARKDHPVWRLM
AN3157.1  	RIRPPEHNRNVIFDVHGVTSIDGSGAQVLYEIVDGYAQAGTRVFFCRVPNR---SVYRMF
          	*:*    *:*::**:*****:****:*** *** .* : *.**:*.* *      *::::

MG09838.1 	ENSGIVEMVGGIDHFVDDVHEALRLTEGPDSGDLPAAS----
NCU09642.1	KRSGIVDLVGGEGHFVSDVGEALKMTEVEQEE----------
FG04242.1 	SSSGIVEMAG-DRHFVNDVEEALRLTEYEESITGESSRHREL
AN3157.1  	QRSGIVERCGGMSHFVGHVDEALKLTDSETRPLSASSA----
          	. ****:  *.  ***..* ***::*:        ::
```
